# Methods for REM Sleep Density Analysis: A Scoping Review

**DOI:** 10.3390/clockssleep5040051

**Published:** 2023-12-14

**Authors:** Tamires Tiemi Kishi, Monica Levy Andersen, Ygor Matos Luciano, Viviane Akemi Kakazu, Sergio Tufik, Gabriel Natan Pires

**Affiliations:** 1 Departamento de Psicobiologia, Federal University of São Paulo, São Paulo 04024-002, Brazil; tamires.tiemi07@gmail.com (T.T.K.);; 2 Sleep Institute, São Paulo, 04020-060, Brazil

**Keywords:** sleep, REM sleep density, polysomnography, depression

## Abstract

Rapid eye movements (REM) sleep density is the parameter proposed to explain the variability in the amount of eye movements during REM sleep. Alterations in REM sleep density have been proposed as a screening criterion for individuals with depression and other mental health conditions, but its accuracy has not been properly evaluated. The lack of consensus and the variability of the methods used to score it reduces the external validity of the results, hindering an adequate analysis of its diagnostic accuracy and clinical applicability. This scoping review aimed to identify and quantify the methods used to score REM sleep density, describing their main characteristics. A literature search was conducted in PubMed, Scopus, PsycInfo, and Web of Science. Only studies with objective measures for REM sleep density analysis in individuals with depression were considered eligible. The final sample comprised 57 articles, covering 64 analyses of REM sleep density. The relative frequency methods were the predominant measurement parameter for analyzing REM sleep density across studies. The most frequently adopted REM estimation unit was the number of REM events followed by mini-epochs containing REM. The most common unit of measurement were frequency/time measures. The results demonstrate that there is no consistency in the methods used to calculate REM sleep density in the literature, and a high percentage of studies do not describe their methods in sufficient detail. The most used method was the number of REM episodes per minute of REM sleep, but its use is neither unanimous nor consensual. The methodological inconsistencies and omissions among studies limit the replicability, comparability, and clinical applicability of REM sleep density. Future guidelines should discuss and include a specific methodology for the scoring of REM sleep density, so it can be consensually implemented in clinical services and research.

## 1. Introduction

The REM sleep stage is characterized by (and named after) the onset of rapid eye movements during sleep. However, being a phasic sleep event, these eye movements are not observed through the entirety of REM sleep, but are rather presented intermittently. REM sleep density is a sleep parameter proposed to account for the variability on the amount of eye movements within REM sleep. It refers to the proportion of REM sleep that actually contains eye movements [1].

Alterations on REM sleep-related parameters (including REM latency, total REM time, and REM sleep density) are often associated with several psychopathologies [2,3,4,5], especially depression, bipolar disorder, and schizophrenia [6,7,8]. However, REM sleep alterations are still not recognized as a proper diagnostic parameter for any of these conditions, as their actual diagnostic accuracy has not been properly clarified [3].

One important practical aspect limiting the applicability of REM sleep density is the lack of consensus on its definitions and on how to score it. Although the current version of the American Academy of Sleep Medicine (AASM) scoring manual [9] describes standards for scoring rapid movements on the electro-oculogram, it does not provide any specific information on how to score and calculate REM sleep density. Consequently, the definition of REM sleep density being used varies considerably from one research group to another. The use of multiple REM sleep density scoring methods reduces the external validity and reproducibility of findings and impairs a proper analysis of its diagnostic accuracy and clinical applicability.

The current scoping review aims at mapping the literature to identify the REM sleep density scoring methods being used, describing its main characteristics.

## 2. Methods

A scoping review is a type of systematic review intended to map and analyze a field of research on a wider perspective, analyzing its publication output and describing its methodological characteristics. The present study is a secondary investigation of another systematic review on the effect of depressive symptoms in REM sleep density (PROSPERO’S protocol number CRD42022345887) and was written according to the Preferred Reporting Items for Systematic reviews and Meta-Analyses (PRISMA), an extension for scoping reviews (PRISMA-ScR) [10]. For a proper reading of the methods and results, we advise for the distinction between the terms REM, REM sleep, and REM sleep density, which have different meanings. “REM” refers to the occurrence of rapid eye movements, “REM sleep” refers to the referred sleep stage, and “REM sleep density” refers to the proportion of eye movements within the REM sleep stage.

### 2.1. Search Strategy and Eligibility Analysis

A bibliographic search was performed at PubMed, Scopus, and Web of Science (complete collection). The search strategy was initially developed for PubMed (as disclosed below), being adapted to the search engine and syntax of the other databases. The search strategies and search results for all databases are presented in the Supplementary Material.

(“REM density” OR (density AND (REM OR “eye movement*” OR “Sleep, REM”[mesh]))) AND (depression[mesh] OR depression[tiab] OR depress*[tiab] OR affective disorders[mesh] OR mood disorders[mesh]).

The search records from each database were exported to Covidence and the exclusion of duplicate records was performed automatically. Each non-duplicated article was screened and evaluated through a 2-stage process: screening of titles and abstracts, followed by full text analysis. In both stages, the articles were analyzed independently according to the inclusion and exclusion criteria by 2 out of 3 independent Reviewers (TTK, VAK, YML), and discrepancies were solved by another Reviewer (GNP). Studies with objective measures for the analysis of REM sleep density in individuals with depression were considered eligible. We excluded systematic or narrative reviews, discussion articles, meta-analyses, case reports, and other types of theoretical articles. There was no language, publication date, or other restrictions.

In the first selection phase (titles and abstracts), the exclusion criteria were applied in the following order: (1) wrong article type (not an original study), (2) wrong population (individuals under 18 years old or animals), and (3) wrong exposure (no depression). In the second selection phase (full texts), the exclusion criteria were applied in the following order: (1) no full text available, (2) wrong comparator/control, (3) no depression, (4) wrong population, (5) non-original article, (6) wrong experimental design, and (7) wrong outcome (no REM sleep density).

### 2.2. Data Extraction

**Figure 1:**
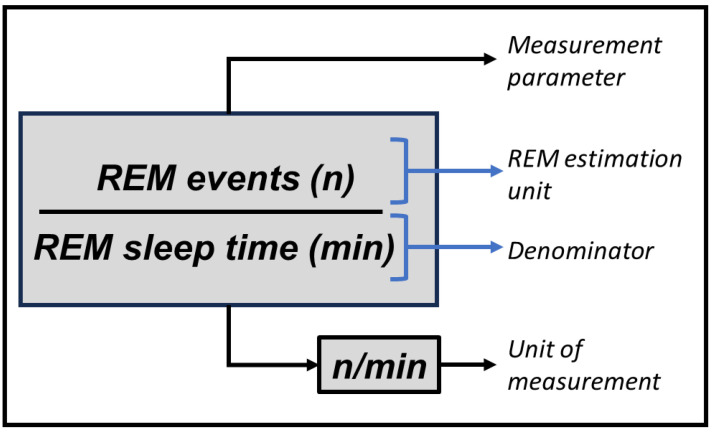
Terminology used to define the methods of REM sleep density analyses. This example details a relative time method, in which the number of REM events is divided by the total REM sleep time. Absolute measurement methods (time or duration) have no denominator. Acronyms: REM: rapid eye movements. N: frequency/number of events. Min: minutes.

Data extraction was conducted by a single Reviewer (TTK) and verified by a second Reviewer (GNP) using a standardized Microsoft Excel data extraction form. For each article, the following variables were extracted:

- Measurement parameter: The parameters by which REM sleep density are measured vary on 2 basic aspects: how REM events are counted and if it is calculated or corrected in relation to some other parameter (as explained in Figure 1). REM events can be measured by either counting the frequency of REM events or determining the total time of REM events. Also, they can be calculated either as absolute measures (when the final parameter is not controlled or compared to any other variable) or relative measures (when they were corrected by some other variable, such as REM sleep stage time or total sleep time). Based on that, each study was categorized as evaluating REM sleep density based on the following alternatives:
  ○ Absolute frequency: total frequency of REM events (or other way to count REM events, such as epochs, mini-epochs, or bursts containing REM).
  ○ Relative frequency: absolute frequency of REM events divided by a denominator.
  ○ Absolute time: total duration of the REM events.
  ○ Relative time: absolute time divided by a denominator.
  ○ Score: Measures that use a score to calculate REM sleep density, based but not directly convertible from frequency or time of REM.
- Method of analysis: Automatic or manual.
- Software: The name of the software used for automatic REM sleep density analysis, when available. It refers specifically to the software used for REM sleep density analyses (rather than to PSG recording or scoring software).
- REM estimation unit: Unit of analysis used to quantify REM events. In relative measurement parameters, it refers to the numerator. Ex.: REM events, mini-epochs with REM or REM bursts.
- Denominator: Denominator used in the measurement. Applicable only to relative measurement parameters. Ex.: total REM sleep time, total number of REM sleep epochs, or total sleep time.
- Unit of measurement: Resulting unit of measurement of REM sleep density. Ex.: REM units per minute, mini-epochs containing REM per minute, etc.
- Size of the mini-epochs: Size of mini-epochs considered in the analysis per REM period (when applicable).
- Eye movements’ definition: Polysomnographic criteria for REM considered in the study.
- Type of polysomnography: Type I (laboratory) or type II (domiciliary) PSG. Types III and IV were not considered as they do not encompass an electro-oculogram, therefore not allowing the quantification of REM events.
- Duration of REM sleep density analysis: Some studies evaluate REM sleep density based on the entire night while others consider shorter analyses (such as the first REM sleep episode).

### 2.3. Data Synthesis and Analyses

As some articles employed 2 or more methods of REM sleep density analysis, the number of analyses and articles might not match exactly and are presented independently whenever applicable. As common for scoping reviews, effect sizes and other summary measures of effect were not calculated. The results are restricted to a descriptive presentation of the data, exploring their quantitative and qualitative aspects (known as “charting analysis”).

## 3. Results

**Figure 2:**
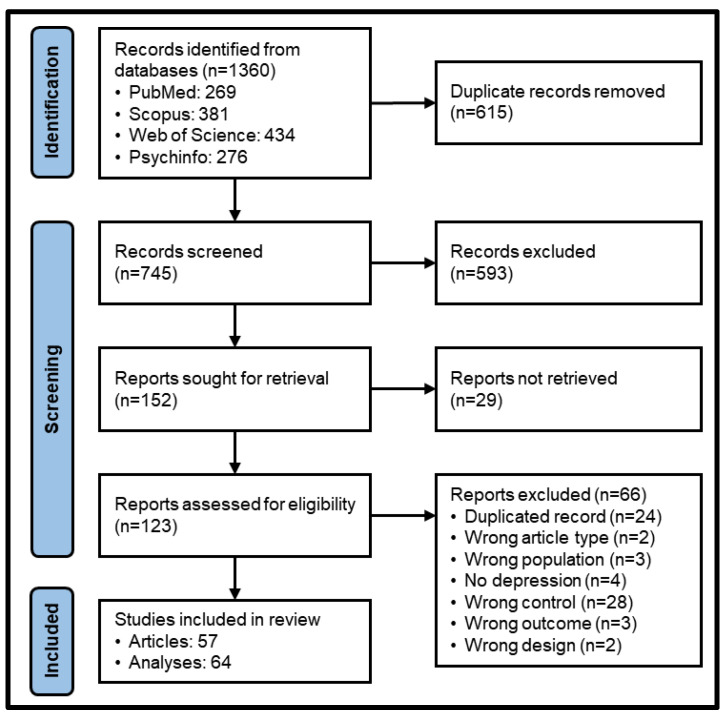
Scoping review flowchart.

The search strategy resulted in 745 non-duplicated records. After screening and eligibility analyses, 57 studies were considered eligible and were included in the final sample, comprising a total of 64 analyses (Table 1). The article selection process is disclosed in Figure 2.

Most of the REM sleep density analyses in our sample were manual (45 analyses—70.31%, 43 articles—91.78%). Among those that used automatic methods (12 analyses—18.75%, 7 articles—12.28%), 2 studies used REMDETK software (4 analyses), 1 used PRANA (1 analysis), 2 used an unspecified algorithm (5 analyses), and 1 used an unspecified semi-automatic algorithm (1 analysis). The remaining ones did not specify how REM sleep density was estimated or calculated (seven analyses—10.93%, seven articles—12.28%). The polysomnographic definitions of eye movements or their scoring criteria were not presented in most studies (52 analyses—81.25%, 50 articles—87.71%), and this information was restricted to the studies using automatic eye movements measurement tools. Even among these, the definition of eye movements was highly variable, the most common being that eye movements are an event with a minimum of 25 microvolts excursion lasting at least 200 msec (7 analyses—10.93%, 2 articles—3.5%).

**Figure 3:**
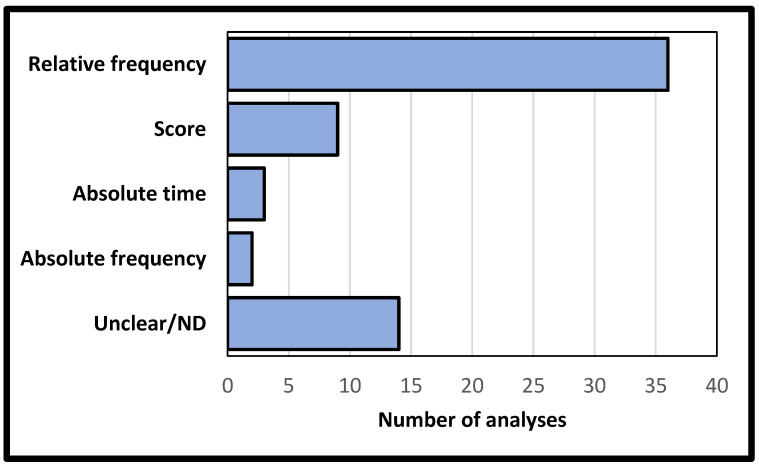
Measurement parameters used for the analysis of REM sleep density. Acronyms: ND: Not disclosed.

The relative frequency methods were the predominant measurement parameter for analyzing REM sleep density across studies (36 analyses—56.25%, 34 articles—59.64%), followed by studies evaluating REM sleep density based on indirect score measures (9 analyses—14.06%, 8 articles—14.04%). A significant number of studies did not provide the adopted measure with sufficient detail to allow classification (12 analyses—18.75%, 12 article—21.05%) (Figure 3).

**Figure 4:**
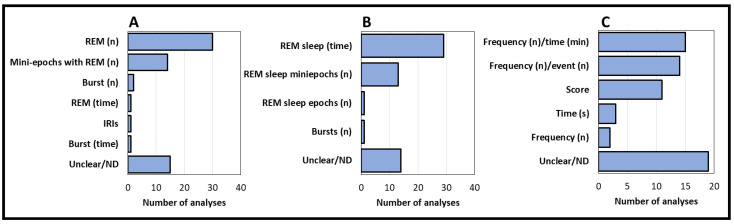
Variables related to the calculation of REM sleep density. (**A**) REM estimation unit. It disclosed the exact way by which REM was estimated. (**B**) Denominator. It refers to the variables used to correct for the amount of REM, being applicable only to relative measurement parameters. (**C**) Unit of measurement. It refers to the resulting unit of measurement of REM sleep density. Acronyms: IRIs: interrapid eye movements interval. Min: minutes. N: frequency/number of events. ND: not disclosed. REM: rapid eye movements. S: seconds.

The most frequently adopted REM estimation unit in the sample was the number of REM events (30 analyses—46.88%, 27 articles—47.37%), followed by mini-epochs containing REM (14 analyses—21.88%, 14 articles—24.56%) (Figure 4A). Among studies adopting the number of mini-epochs containing REM as the estimation unit, 3 s mini-epochs were the most common (13 analyses—20.31%, 13 articles—22.81%), while epochs of 2, 5, and 20 s were employed by 1 analysis each. Regarding the denominator applied, we noticed a predominance of total REM sleep time (29 analyses—45.31%, 29 articles—50.87%), followed by the number of REM sleep mini-epochs (13 analyses—20.31%, 13 articles—22.81%) (Figure 4B).

For the unit of measurement, most studies did not describe or allow us to deduct which unit of measurement was used to evaluate REM sleep density assessment. Among the cases from which this information could be extracted, frequency/time measures, especially REM units per minute of REM sleep, were the most frequent unit of measurement used (15 analyses—23.44%, 15 articles—26.32%). Following, frequency/event measures, especially mini-epochs containing REM divided by the total number of REM sleep mini-epochs, were the second most used unit of measurement (14 analyses—21.8%, 14 articles—24.56%) (Figure 4C).

Most studies presented the REM sleep density analysis related both to the first REM sleep episode and to the whole night (29 analyses—45.31%, 26 articles—45.61%). A similar number of studies presented the results related to the entire night only (26 analyses—40.62%, 23 articles—40.35%), while few of them restricted the results to the first REM sleep episode (4 analyses—6.25%, 4 articles—7.01%) (Figure 4). Only 1 analysis in our sample was performed using type II polysomnography, while the other analyses were performed using type I polysomnography (63 analyses—98.43%, 56 articles—98.24%).

## 4. Discussion

Variations on the scoring and diagnostic criteria are demonstrated in the literature as a factor that affects not only the replicability of research, but also may generate variations in prevalence estimates. As an example, differences on the hypopnea scoring criteria might overestimate the prevalence of moderate to severe obstructive sleep apnea in about 50% [67]. In view of the above, it is essential that REM sleep density analysis methods be standardized to provide reliable results and comparisons.

Our results showed that there is a high variability in the methodologies and calculations applied to estimate REM sleep density, and a high percentage of studies do not even describe their methods in sufficient detail. The most used method was the number of REM episodes per minute of REM sleep, but its use is neither unanimous nor consensual. The methodological inconsistencies and omissions among studies are an important drawback on the comparability and clinical applicability of REM sleep density. Another relevant aspect is the variability of the software and algorithms created to perform the automatic analysis of REM sleep density. Most of them do not describe the scoring parameters used, and their validation and diagnostic accuracy in comparison to human scorers have not been tested.

To date, the best definition of eye movements in a PSG recording is provided by the AASM scoring manual (AASM, 2023 [9]), which defines it as “conjugate, irregular, sharply peaked eye movements with an initial deflection usually lasting <500 msec”. However, none of the included studies in our sample met or directly mentioned these criteria. Even considering that there is a proper definition of REM, no manual provides guidance for the calculation of REM sleep density whatsoever. It is still necessary to standardize the methods of REM sleep density analysis for future research to generate more reliable and accurate data.

Although we highlight that there is no consensus on the evaluation of REM sleep density, our study is not able to approach important practical problems. First, by the current results, we are not able to estimate the level of discrepancies among the different REM sleep density calculation methods. It is very likely that different methods would lead to different estimates even when applied to the same PSG recording. However, neither the magnitude of this discrepancy nor how much one method under or overestimates REM sleep density over another can be determined by our methodological approach. We also cannot determine which is the best method for calculating REM sleep density. Solving these issues would require analyzing polysomnographic data from a clinically characterized sample and evaluating the accuracy of different methods to estimate REM sleep density on predicting specific outcomes.

There is growing evidence regarding the relevance of REM sleep density both as a prognostic and as a diagnostic measure. REM-sleep dysregulation has previously been associated with depression and has been proposed as a potential endophenotype of depression [3,68,69]. It has also been considered as a marker of vulnerability for depressive disorder in a high-risk group, even in patients who are not undergoing a period of clinically observable depressive symptoms [41]. In such cases, elevated REM sleep density during the first REM episode was stable over a 4-year period and demonstrated that elevated REM sleep density indeed represents a valid neurobiological marker of vulnerability to depression [33,42,70]. Changes in REM sleep density are also associated with patient response to treatment, but with discrepant results. Lechinger et al. described that the response to antidepressant pharmacotherapy among patients with moderate to severe depression is better in those with high baseline REM sleep density [71]. Conversely, Clark et al. demonstrated a more robust response to the antidepressant acute effects of sleep deprivation among those with lower baseline REM sleep density [16]. Although these data reinforce the possible relevance of REM sleep density as an important polysomnographic measure, with implications on diagnosis, prognosis, and response to treatment, its practical implementation would depend on having standardized measures to analyze, score, and report it.

As a limitation of our study, it shall be mentioned that this is a secondary analysis of a systematic review of REM sleep density alterations in individuals with depressive symptoms. We acknowledge that REM sleep density is not exclusively related to depression and evaluating it may be relevant under the scope of other mood disorders (bipolar disorder and mania included), other mental health conditions (such as schizophrenia), and sleep disorders involving REM sleep alterations (such as narcolepsy and REM sleep behavior disorder). Although this scoping review was based on studies specifically about depression, we believe this is not likely to influence the results or decrease the reliability of these findings, due to two main reasons. First, depression is the condition most closely related to REM sleep density alterations. Second, we believe that the same inconsistencies on the methods used to quantify REM sleep density would have been achieved even if our search were broader to encompass bipolar disorder and other conditions.

In conclusion, our study demonstrates that there is no consistency on the methods used to calculate REM sleep density in the current literature. Despite the potential usefulness of this measure for the screening and differential diagnosis of medical and psychiatric conditions, we are not able to define which is the best method to quantify it. Based on that, we suggest that future scoring manuals or tasks forces on sleep scoring guidelines discuss and include specific methodology for the calculation of REM sleep density, so it can be consensually implemented in clinical services and research.

## Figures and Tables

**Table 1:** Descriptions and characterization of the included studies.

Study	Analysis	PSG Type	Method of Analysis	Software	Measurement Parameter	REM Estimation Unit	Denominator	Unit of Measurement	Duration of REM Sleep Density Analysis	Mini-Epoch Size
Ansseau et al., 1985 [11]	1	I	Manual	NA	Relative frequency	REM (n)	REM sleep (time)	Frequency (n)/time (min)	Whole night	NA
Antonijevic et al., 2003 [12]	1	I	Manual	NA	Unclear/ND	Unclear/ND	Unclear/ND	Unclear/ND	Whole night	NA
Asaad et al., 2002 [13]	1	I	Manual	NA	Relative frequency	Mini-epochs with REM (n)	REM sleep epochs (n)	Unclear/ND	Both	3 s
Benson et al., 1993 [14]	1	I	Automatic	REMDTEK	Relative frequency	REM (n)	REM sleep (time)	Frequency (n)/time (min)	Both	NA
Benson et al., 1993 [14]	2	I	Automatic	REMDTEK	Absolute time	Burst (time)	NA	Time (s)	Both	NA
Benson et al., 1993 [14]	3	I	Automatic	REMDTEK	Relative frequency	REM (n)	Burst (n)	Frequency (n)/event (n)	Both	NA
Cartwright et al., 1998 [15]	1	I	Manual	NA	Unclear/ND	Unclear/ND	Unclear/ND	Unclear/ND	First REM episode	NA
Clark et al., 2000 [16]	1	I	Manual	NA	Score	REM (n)	REM sleep (time)	Score	Whole night	NA
Clark et al., 1998 [17]	1	I	Manual	NA	Score	REM (n)	REM sleep (time)	Score	Whole night	NA
Douglass et al., 1992 [18]	1	I	Automatic	REMDTEK	Relative frequency	IRIs (Interrapid EM Interval)	REM sleep (time)	Frequency (n)/time (min)	Whole night	NA
Dow et al., 1996 [19]	1	I	Manual	NA	Score	REM (n)	REM sleep (time)	Score	Whole night	NA
Dykierek et al., 1998 [20]	1	I	Manual	NA	Relative frequency	Mini-epochs with REM (n)	REM sleep mini-epochs (n)	Frequency (n)/event (n)	Both	3 s
Friess et al., 2008 [21]	1	I	Manual	NA	Relative frequency	Mini-epochs with REM (n)	REM sleep mini-epochs (n)	Frequency (n)/event (n)	Both	3 s
Gann et al., 2004 [22]	1	I	ND	ND	Unclear/ND	Unclear/ND	Unclear/ND	Unclear/ND	Both	NA
Gann et al., 1992 [23]	1	I	Manual	NA	Unclear/ND	Unclear/ND	Unclear/ND	Unclear/ND	Both	3 s
Gillin et al., 1981 [24]	1	I	Manual	NA	Unclear/ND	Unclear/ND	Unclear/ND	Unclear/ND	Both	NA
Gillin et al., 1979 [25]	1	I	Manual	NA	Score	REM (n)	REM sleep (time)	Score	Whole night	NA
Gillin et al., 1996 [26]	1	I	Manual	NA	Score	REM (n)	REM sleep (time)	Score	Whole night	NA
Hudson et al., 1992 [27]	1	I	Manual	NA	Relative frequency	REM (n)	REM sleep (time)	Frequency (n)/time (min)	Both	NA
Hudson et al., 1987 [28]	1	I	Manual	NA	Relative frequency	REM (n)	REM sleep (time)	Frequency (n)/time (min)	First REM episode	NA
Jones et al., 1985 [29]	1	I	ND	ND	Unclear/ND	Unclear/ND	Unclear/ND	Unclear/ND	First REM episode	NA
Kwon et al., 2019 [30]	1	I	Automatic	Semi-automatic algorithm	Relative frequency	Mini-epochs with REM (n)	REM sleep mini-epochs (n)	Frequency (n)/event (n)	Whole night	3 s
Lauer et al., 1990 [31]	1	I	Manual	NA	Unclear/ND	Unclear/ND	Unclear/ND	Unclear/ND	Both	NA
Lauer et al., 1991 [32]	1	I	Manual	NA	Relative frequency	Mini-epochs with REM (n)	REM sleep mini-epochs (n)	Frequency (n)/event (n)	Both	3 s
Lauer et al., 1995 [33]	1	I	Manual	NA	Relative frequency	Mini-epochs with REM (n)	REM sleep mini-epochs (n)	Frequency (n)/event (n)	Both	3 s
Linkowski et al., 1986 [34]	1	I	Manual	NA	Relative frequency	Unclear/ND	REM sleep (time)	Unclear/ND	Whole night	NA
Liscombe et al., 2002 [35]	1	I	ND	ND	Unclear/ND	Unclear/ND	Unclear/ND	Unclear/ND	Unclear/ND	NA
Luik et al., 2015 [36]	1	II	Automatic	PRANA	Relative frequency	REM (n)	REM sleep (time)	Frequency (n)/time (min)	Whole night	NA
Mcnamara et al., 1984 [37]	1	I	Manual	NA	Relative frequency	REM (n)	REM sleep (time)	Unclear/ND	Both	NA
Mellman et al., 1997 [38]	1	I	Manual	NA	Relative frequency	REM (n)	REM sleep (time)	Frequency (n)/time (min)	Whole night	NA
Mendlewicz et al., 1991 [39]	1	I	Manual	NA	Relative frequency	REM (n)	REM sleep (time)	Frequency (n)/time (min)	Whole night	NA
Mendlewicz et al., 1984 [40]	1	I	Manual	NA	Relative frequency	REM (n)	REM sleep (time)	Score	Whole night	NA
Modell et al., 2002 [41]	1	I	ND	ND	Unclear/ND	Unclear/ND	Unclear/ND	Unclear/ND	Both	NA
Modell et al., 2005 [42]	1	I	Manual	NA	Relative frequency	Mini-epochs with REM (n)	REM sleep mini-epochs (n)	Frequency (n)/event (n)	Both	3 s
Moore et al., 1998 [43]	1	I	Manual	NA	Score	REM (n)	REM sleep (time)	Score	Both	NA
Motivala et al., 2005 [44]	1	I	Manual	NA	Score	REM (n)	REM sleep (time)	Score	First REM episode	NA
Pasternak et al., 1994 [45]	1	I	ND	ND	Unclear/ND	Unclear/ND	Unclear/ND	Unclear/ND	Unclear/ND	NA
Pawlowski et al., 2017 [1]	1	I	Automatic	Unspecified algorithm	Relative frequency	Mini-epochs with REM (n)	REM sleep mini-epochs (n)	Frequency (n)/event (n)	Whole night	3 s
Poland et al., 1997 [46]	1	I	Manual	NA	Relative frequency	REM (n)	REM sleep (time)	Frequency (n)/time (min)	Both	NA
Reynolds et al., 1985 [47]	1	I	Manual	NA	Relative frequency	REM (n)	REM sleep (time)	Unclear/ND	Both	NA
Reynolds et al., 1987 [48]	1	I	Manual	NA	Relative frequency	REM (n)	REM sleep (time)	Unclear/ND	Both	NA
Riemann et al., 1994 [49]	1	I	Manual	NA	Unclear/ND	Unclear/ND	Unclear/ND	Unclear/ND	Unclear/ND	NA
Riemann et al., 1994 [50]	1	I	Manual	NA	Relative frequency	Mini-epochs with REM (n)	REM sleep mini-epochs (n)	Frequency (n)/event (n)	Both	3 s
Riemann et al., 1994 [51]	1	I	Manual	NA	Relative frequency	Mini-epochs with REM (n)	REM sleep mini-epochs (n)	Frequency (n)/event (n)	Both	3 s
Rotenberg et al., 2000 [52]	1	I	Manual	NA	Relative frequency	REM (n)	REM sleep (time)	Frequency (n)/time (min)	Both	NA
Rotenberg et al., 1997 [53]	1	I	Manual	NA	Relative frequency	REM (n)	REM sleep (time)	Frequency (n)/time (min)	Whole night	NA
Shipley et al., 1992 [54]	1	I	Manual	NA	Relative frequency	REM (n)	REM sleep (time)	Score	Whole night	NA
Sitaram et al., 1982 [55]	1	I	Manual	NA	Score	REM (n)	REM sleep (time)	Score	Both	NA
Sitaram et al., 1984 [56]	1	I	Manual	NA	Unclear/ND	Unclear/ND	Unclear/ND	Unclear/ND	Both	NA
Steiger et al., 1994 [57]	1	I	Manual	NA	Relative frequency	Mini-epochs with REM (n)	REM sleep mini-epochs (n)	Frequency (n)/event (n)	Unclear/ND	3 s
Talbot et al., 2009 [58]	1	I	Manual	NA	Relative frequency	Mini-epochs with REM (n)	REM sleep mini-epochs (n)	Frequency (n)/event (n)	Whole night	5 s
Taylor et al., 1999 [59]	1	I	Automatic	ND	Relative frequency	REM (n)	REM sleep (time)	Frequency (n)/time (min)	Whole night	NA
Thase et al., 1997 [60]	1	I	Manual	NA	Relative frequency	REM (n)	REM sleep (time)	Frequency (n)/time (min)	Unclear/ND	NA
Thase et al., 1994 [61]	1	I	Manual	NA	Relative frequency	REM (n)	REM sleep (time)	Frequency (n)/time (min)	Whole night	NA
Waller et al., 1989 [62]	1	I	ND	ND	Unclear/ND	Unclear/ND	Unclear/ND	Unclear/ND	Both	NA
Wichniak et al., 2002 [63]	1	I	Manual	NA	Relative frequency	Mini-epochs with REM (n)	REM sleep mini-epochs (n)	Frequency (n)/event (n)	Both	2 s
Wichniak et al., 2002 [63]	2	I	Manual	NA	Score	REM (time)	NA	Score	Both	20 s
Wichniak et al., 2002 [63]	3	I	Manual	NA	Relative frequency	REM (n)	REM sleep (time)	Frequency (n)/time (min)	Both	NA
Wichniak et al., 2000 [64]	1	I	Manual	NA	Relative frequency	Mini-epochs with REM (n)	REM sleep mini-epochs (n)	Frequency (n)/event (n)	Whole night	3 s
Youssef et al., 2011 [65]	1	I	ND	ND	Unclear/ND	Unclear/ND	Unclear/ND	Unclear/ND	Whole night	NA
Zarcone et al., 1983 [66]	1	I	Automatic	Unspecified algorithm	Absolute frequency	REM (n)	NA	Frequency (n)	Whole night	NA
Zarcone et al., 1983 [66]	2	I	Automatic	Unspecified algorithm	Absolute frequency	Burst (n)	NA	Frequency (n)	Whole night	NA
Zarcone et al., 1983 [66]	3	I	Automatic	Unspecified algorithm	Absolute time	REM (n)	NA	Time (s)	Whole night	NA
Zarcone et al., 1983 [66]	4	I	Automatic	Unspecified algorithm	Absolute time	Burst (n)	NA	Time (s)	Whole night	NA

Acronyms: n: frequency/number of events. Min: minutes. NA: Not applicable. ND: not disclosed. REM: rapid eye movements. s: seconds.

## Data Availability

Available upon request.

## Supplementary Materials

The following supporting information can be downloaded at: https://www.mdpi.com/article/10.3390/clockssleep5040051/s1, Search strategies.
