# Supplementary material for: Methods for REM Sleep Density Analysis: A Scoping Review
Source: Clocks Sleep. 2023 Dec 14;5(4):793–805. doi: 10.3390/clockssleep5040051 (PMC10742531; doi:10.3390/clockssleep5040051)
Supplement: Supplementary file 1 [file clockssleep-05-00051-s001.zip › clockssleep-2685598-supplementary.pdf]

METHODS FOR REM SLEEP DENSITY ANALYSIS: A SCOPING REVIEW

SEARCH STRATEGY

Systematic review and meta-analysis – REM sleep density in people with depression

Total (Pubmed, Scopus, WoS, PsycInfo): 1,360

Total (deduplicated): 615

- **PubMed:** 269 records
  - Search date: 13/07/2022
  - ("REM density" OR (density AND (REM OR "eye movement*" OR "Sleep, REM"[mesh]))) AND (depression[mesh] OR depression[tiab] OR depress*[tiab] OR affective disorders[mesh] OR mood disorders[mesh])

- **Scopus**: 381 records
  - Search date: 13/07/2022
  - (TITLE-ABS-KEY ("REM density" OR (density AND (REM OR "eye movement*"))) AND TITLE-ABS-KEY (depress* OR "affective disorder*" OR "mood disorder*"))

- **Web of Science** (all databases): 434 records
  - Search date: 13/07/2022
  - TS=(("REM density" OR (density AND (REM OR "eye movement*"))) AND (depress* OR "affective disorder*" OR "mood disorder*"))

- **PsycInfo**: 276 records
  - Search date: 13/07/2022
  - ("REM density" OR (density AND (REM OR "eye movement*"))) AND (depress* OR "affective disorder*" OR "mood disorder*")
